# *Helicobacter pylori* DNA methyltransferases and the epigenetic field effect in cancerization

**DOI:** 10.3389/fmicb.2014.00115

**Published:** 2014-03-26

**Authors:** Ramakrishnan Sitaraman

**Affiliations:** Department of Biotechnology, TERI UniversityNew Delhi, India

**Keywords:** *Helicobacter pylori*, epigenetic field effect, gastric cancer, DNA methyltransferase, DNA methylation, host-pathogen interactions, gastric microbiota

## Introduction

*Helicobacter pylori*, a Gram-negative, microaerophilic bacterium, has co-existed with humans beings as a prominent member of their gastric microbiota for approximately 10^5^ years (Moodley et al., 2012). It infects approximately half the world's population, and most infected individuals are asymptomatic, but histologically exhibit superficial gastritis (The EUROGAST Study Group, 1993). Only a minority of infected individuals develop gastric or duodenal ulcers that necessitate treatment. Prolonged inflammation caused by chronic (often lifelong) infection predisposes a small fraction of infected individuals to develop gastric adenocarcinoma or lymphoma of the mucosa-associated lymphoid tissue (MALT lymphoma) (Passaro et al., 2002). Unfortunately, the prognosis for cases of gastric cancer is very poor, with 5-year survival rates being lower than 15% (Peek and Blaser, 2002).

A mechanism for carcinogenesis ensuing from *H. pylori*-triggered inflammation was first proposed by Pelayo Correa (Correa, 1992; Correa and Piazuelo, 2012). Briefly, chronic inflammation causes superficial gastritis that progresses over time to multifocal atrophic gastritis (MAG), characterized by the destruction of gastric glands. This is followed by intestinal metaplasia, wherein gastric epithelium undergoes an “epithelial-mesenchymal transition” and begins to exhibit an intestinal phenotype. The subsequent stage consists of dysplasia culminating in invasive carcinoma, which completes the “pre-cancerous cascade.” The final outcome is also dependent on host and pathogen genotypes, as well as environmental factors such as socioeconomic indicators, a high-salt diet, low fruit/vegetable intake and smoking (Khalifa et al., 2010). Most notably, *H. pylori* is the sole bacterium to be classified by the WHO as a class I carcinogen (IARC Working Group on the Evaluation of Carcinogenic Risks to Humans, 1994).

## A mechanism for epigenetic field cancerization by *H. pylori* DNA MTases

Field cancerization is a concept first proposed in 1953 in the context of oral stratified squamous epithelium (Slaughter et al., 1953), and subsequently extended to other tissues. Briefly, upon exposure to a carcinogen at sufficient intensity for a significant duration, grossly normal-looking tissue near tumor sites suffers microscopic (essentially, molecular) changes that eventually result in carcinogenesis. Aberrant methylation of cytosine residues within CpG islands (CGI) in genomic DNA has been reported in a variety of cancers, including gastric cancer (Laird and Jaenisch, 1994; Laird, 2005). This is an epigenetic change that could contribute to cancer development by the process of field cancerization (Ramachandran and Singal, 2012). Chronic *H. pylori* infection in humans is associated with hypermethylation of promoter sequences of different categories of genes, resulting in downregulation of transcription. Some of these are: *CDH1* that codes for E-cadherin, a transmembrane glycoprotein involved in maintaining epithelial integrity (Chan et al., 2003); *GATA4* and *GATA5* encoding transcription factors (Wen et al., 2010); and *TFF2* (encoding trefoil factor 2) (Peterson et al., 2010) and *FOXD3* (encoding a forkhead box transcriptional regulator) that are tumor suppressors (Cheng et al., 2013). However, one unexplored possibility is that one or more of the several functional DNA methyltransferases (MTases) of *H. pylori* could enter host cells and methylate their recognition sequences in chromosomal DNA in an unregulated manner. The result would be the creation of an epigenetic field of cancerization.

### The numerous DNA MTases of *H. pylori*

DNA MTases are sequence-specific DNA-binding enzymes that methylate adenine or cytosine residues in the context of their cognate recognition sequences using S-adenosylmethionine as the methyl donor, and are widespread among prokaryotes (Roberts et al., 2010). Depending on the enzyme in question, a methyl group may be added at the *N*^6^ position in adenine forming *N*^6^-methyladenine (m6A) or the *N*^4^ or *C*^5^ positions in cytosine forming *N*^4^-methylcytosine (m4C) or *C*^5^-methylcytosine (m5C) respectively. DNA methylation of regulatory sequences is known to result in changes in gene expression in a wide variety of organisms, both prokaryotes and eukaryotes. Therefore, should DNA MTases encoded by pathogens gain entry into host cells by specific or non-specific means, there is a strong possibility that they could modify host regulatory DNA sequences (nuclear, and perhaps even organellar), making the process at least partially inflammation-independent.

Several pathogens, including *H. pylori*, are known to introduce virulence factors into eukaryotic host cell by a variety of mechanisms. Recently, a type I DNA methyltransferase subunit (HsdM) of *Klebsiella pneumoniae* was found to have a nuclear localization signal (NLS). When expressed in in the COS-1 (African green monkey kidney) cell line, HsdM localized to the nucleus. Surprisingly, HsdM was capable of methylating DNA even in the absence of the specificity subunit (HsdS), albeit at much lower levels (Lee et al., 2009). A recent study demonstrated that a transposase (Tnp) of *Acinetobacter baumanii* was targeted to the nuclei of A549 (a human lung carcinoma) and COS-7 (African green monkey kidney) cell lines, and that this resulted in specific CpG methylation of the *CDH1* (E-cadherin) promoter (Moon et al., 2012).

A survey of the database of restriction enzymes (REBASE; http://rebase.neb.com) indicates that *H. pylori* encodes a noticeably large number of DNA MTases, known or putative—ranging from 25 in the strain SouthAfrica7 to 37 in strain Puno135. Very few prokaryotes encode such a large number of DNA MTases or restriction-modification (R-M) systems. A majority of the predicted/known DNA MTases encoded by *H. pylori*, both adenine- and cytosine-specific, are type II enzymes (http://tools.neb.com/~vincze/genomes/index.php?page=H). In this class of DNA MTases, the functions of sequence-specific DNA binding and DNA methylation are carried out by the same protein, and do not require any accessory protein factors for full activity. DNA transfer experiments between *H. pylori* strains clearly demonstrated sequence-specific DNA methylation in cell extracts (Donahue et al., 2000). Several studies have indicated that many of the DNA MTases encoded by the *H. pylori* genome are expressed and functional (Vitkute et al., 2001; Takata et al., 2002; Vale and Vítor, 2007; Kumar et al., 2012a), and can affect *H. pylori* protein expression in a strain-specific manner (Donahue et al., 2002; Takata et al., 2002; Kumar et al., 2012a; Vitoriano et al., 2013).

### Entry of *H. pylori* DNA MTases into host cells

There are at least three mutually non-exclusive routes by which DNA MTases could gain entry into host cells. Firstly, while *H. pylori* is predominantly extracellular, studies have indicated that it may be a facultatively intracellular as well (Kwok et al., 2002; Necchi et al., 2007; Liu et al., 2012). As a chronic pathogen, its intracellular persistence could conceivably result in the DNA MTases gaining access into the host cell cytoplasm. Secondly, *H. pylori* is also known to release membrane vesicles containing cellular proteins, and it is possible that DNA MTases could be transported into to the host cytoplasm in these vesicles. However, a recent proteomic study of *H. pylori* vesicles failed to detect any DNA MTases in them, indicating that this is unlikely, but it is possible that growth of *H. pylori* on plates or in broth might not correspond to the situation *in vivo* (Olofsson et al., 2010). Thirdly, many *H. pylori* strains encode components of a type IV secretion system (termed the *cag* pathogenicity island, *cag* PAI) that is capable of translocating a protein, CagA (Odenbreit et al., 2000), and peptidoglycan (Viala et al., 2004) into host cells. Presently, there is no conclusive data on whether or not DNA MTases or other cell components could be similarly translocated, though a recent computational prediction using indicates that 1–3 DNA MTases could translocated by the *cag*PAI (Wang et al., 2014), based on the model underlying the prediction.

Regardless of the mechanism by which bacterial proteins might enter the host cells, the fact remains that none of the known DNA MTases (or restriction endonucleases) of *H. pylori* possess any recognizable nuclear localization signals, so that the actual mechanism of nuclear translocation required for DNA methylation, if it happens, is still open to question.

### Inferences from studies in the Mongolian gerbil (*meriones unguiculatus*) model

Humans are the only known natural host for *H. pylori*. However, *H. pylori*-infected Mongolian gerbils reproducibly develop gastric adenocarcinoma upon oral *N*-methyl-*N*-nitosourea administration, and have therefore been used in animal studies for more than 15 years now (Watanabe et al., 1998). Niwa *et al*. have used this model system to examine DNA methylation in gastric cancer in detail over a duration of up to a year (Niwa et al., 2013, 2010). In their earlier study, they first demonstrated that carcinogenesis is accompanied by hypermethylation of promoters, and that cyclosporin A (CsA), an anti-inflammatory agent, does not interfere with bacterial colonization of the animals, but abrogates DNA hypermethylation significantly. Their studies demonstrated that the inflammatory response to *H. pylori* infection in Mongolian gerbils is associated with an increase in DNA methylation in gastric epithelial cells (GECs). This was taken by them to imply that methylation is not directly attributable to any bacterial effectors such as CagA or DNA MTases (Niwa et al., 2010). However, the same study also observed an unexpected decrease in the transcription levels of the host DNA MTases (Dnmts) in the GECs of infected gerbils compared to uninfected controls. Is it possible that cellular Dnmts are down-regulated in response to the chronic burden of a large number of bacterial DNA MTases, and the significant association of *H. pylori* with cancer development is due, in some part, to its large complement of DNA MTases? An additional fact to consider is that bacterial DNA adenine MTases, depending on their specificity, could modify adenine residues in regulatory regions in the DNA. More importantly, the specificity of some adenine MTases may also be relaxed, resulting in cytosine methylation at the *N*^4^ position (Jeltsch et al., 1999). A cytosine DNA MTase of *H. pylori* (M.HpyAVIB) was found to exhibit relaxed specificity upon mutation (Kumar et al., 2012b). Lastly, given that adenine methylation is not routinely examined in studies targeting promoter hypermethylation in humans on the basis of the very low incidence of m6A in mammalian DNA, it may well have been missed in studies concentrating on CGI methylation.

## Conclusions

While the link between viruses and cancer has been extensively researched, it is notable that *Helicobacter pylori* has remained the best-studied and, for nearly two decades, the sole bacterial pathogen systematically linked with any type of cancer in clinical practice. Some bacterial effector molecules associated with carcinogenesis, such as CagA and VacA, have been studied in great detail. Owing to a combination of unique characteristics—encoding a large number of functional DNA MTases, lifelong persistence in the host and facultative intracellularity—*H. pylori* may well be a unique member of the stomach microbiota that affects its host in unforeseen ways. The investigation of the effects of the entry of DNA MTases (and restriction endonucleases, including methylation-dependent restriction enzymes, that can cause DNA breaks) and other proteins of the microbiome into host cells has the potential to uncover novel interactions between evolutionarily disparate species. More generally, it is possible that these proteins are effectors of inter-specific epigenetic signals, that perhaps enable commensals, symbionts and pathogens to adapt to their ecological niches by modulating host gene expression. While housekeeping DNA MTases (e.g., the Dam methylase of *E. coli*) of bacteria, pathogenic or non-pathogenic, are known to be important for bacterial viability (Marinus and Casadesus, 2009), the role of bacterial DNA MTases in infectious diseases and importantly, in the evolution and maintenance of host-microbiome interactions remains unclear, and perhaps merits fresh consideration in terms of the epigenetic modulation of host physiology.

### Conflict of interest statement

Work on *Helicobacter pylori* phosopholipases in my laboratory is supported by a grant from the Department of Biotechnology, Government of India (sanction order number BT/PR11740/BRB/10/683/2008).
